# Removal of Pharmaceuticals and Personal Care Products (PPCPs) by Free Radicals in Advanced Oxidation Processes

**DOI:** 10.3390/ma15228152

**Published:** 2022-11-17

**Authors:** Jiao Jiao, Yihua Li, Qi Song, Liujin Wang, Tianlie Luo, Changfei Gao, Lifen Liu, Shengtao Yang

**Affiliations:** 1 Key Laboratory of Pollution Control Chemistry and Environmental Functional Materials for Qinghai-Tibet Plateau of the National Ethnic Affairs Commission, School of Chemistry and Environment, Southwest Minzu University, Chengdu 610041, China; 2 State of Environmental Protection Key Laboratory of Synergetic Control and Joint Remediation for Soil & Water Pollution, College of Ecology and Environment, Chengdu University of Technology, Chengdu 610059, China; 3 School of Environmental and Material Engineering, Yantai University, Yantai 264005, China; 4 Key Laboratory of Industrial Ecology and Environmental Engineering, Ministry of Education, School of Ocean Science and Technology, Dalian University of Technology, Panjin 124221, China

**Keywords:** pharmaceuticals and personal care products, advanced oxidation processes, free radicals, water treatment

## Abstract

As emerging pollutants, pharmaceutical and personal care products (PPCPs) have received extensive attention due to their high detection frequency (with concentrations ranging from ng/L to μg/L) and potential risk to aqueous environments and human health. Advanced oxidation processes (AOPs) are effective techniques for the removal of PPCPs from water environments. In AOPs, different types of free radicals (HO·, SO_4_·^−^, O_2_·^−^, etc.) are generated to decompose PPCPs into non-toxic and small-molecule compounds, finally leading to the decomposition of PPCPs. This review systematically summarizes the features of various AOPs and the removal of PPCPs by different free radicals. The operation conditions and comprehensive performance of different types of free radicals are summarized, and the reaction mechanisms are further revealed. This review will provide a quick understanding of AOPs for later researchers.

## 1. Introduction

Pharmaceutical and personal care products (PPCPs) are attracting increasing concern [1,2,3] due to the fact that they have been extensively detected in aqueous environments, solids and sediments [4,5,6,7,8,9,10]. PPCPs are defined as widespread chemicals including pharmaceuticals (such as hormones, antibiotics, antidepressants, non-steroidal anti-inflammatory drugs, and lipid regulators) and personal care products (such as preservatives, disinfectants, fragrances, and sunscreens) [2,11]. PPCPs are widely used in high quantities throughout the world, and are known to be released into aquatic environments from multiple discharges, including domestic wastewater, pharmaceutical wastewater [11], daily washing, swimming, excreting after human ingestion [12], livestock, aquaculture and households (excretion and littering) [13]. Meanwhile, in terms of household medicine, the inappropriate disposal of pharmaceutical products could adversely infect the environment and increase the risk of accidental poisoning [14]. It was revealed that domestic sewage was the primary source of PPCP emissions in the surface water of China [15,16]. These pollutants, along with their transformed intermediate products, have been prevalent in most environmental matrices [17].

**Figure 1:**
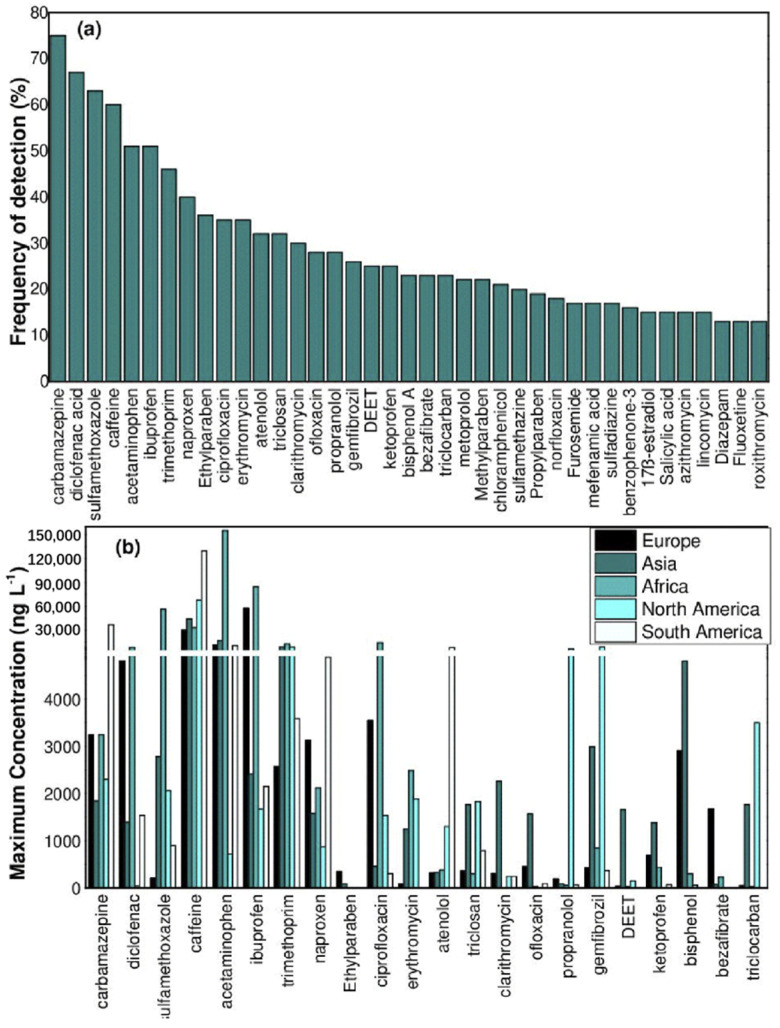
Detection results of PPCPs in surface water. (**a**) Detection frequencies of 104 articles; (**b**) the maximum concentrations (ng/L) of the most detected PPCPs in each analyzed continent. Reproduced with permission from [23].

**Figure 2:**
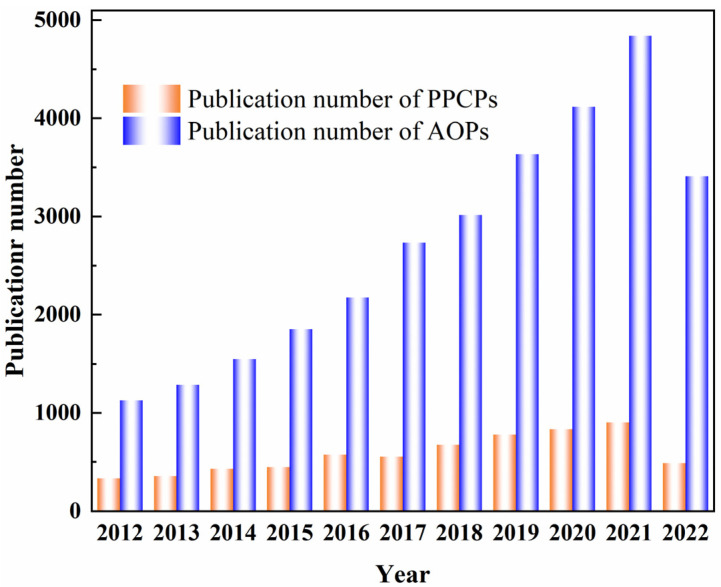
Number of publications on PPCPs and AOPs from 2012 to 2022 (topic keywords “pharmaceutical and personal care products” and “advanced oxidation processes” searched from web of science), data updated by 28 September 2022.

To evaluate the per-capita emission rates of some PPCPs, in Korea, Subedi et al. [18] found that the per-capita emission rates of triclocarban and acetaminophen (ACE) were 158 and 59 μg/capita/day, respectively. In the long run, trace concentrations of 1 ng/L~100 μg/L of PPCPs in aqueous environments pose potential risks to animals and human health [19]. The detection results of partial PPCPs in various aquatic environments from different countries were summarized in Table 1. PPCPs have been widely detected in surface water, groundwater, and even drinking water, with concentrations ranging from ng/L to mg/L. Additionally, the concentrations of PPCPs in sediment and soil are at the level of mg/kg [19,20,21,22]. Recently, Chaves et al. [23] revealed the frequencies of detection of PPCPs in surface water with 104 articles (Figure 1a). Among them, carbamazepine (CBZ), diclofenac (DCF), sulfamethoxazole (SMX), caffeine (CAF), ACE and ibuprofen (IBP) have the highest detection frequency. The maximum concentrations (ng/L) of the most frequent PPCPs in each analyzed continent are shown in Figure 1b. Although the concentrations of PPCPs are not enough to cause acute toxicity to humans or animals, most of the PPCPs will gradually accumulate in the aqueous organisms for their refractory properties, causing potential threats to human health [24,25,26]. Meanwhile, the ubiquitous presence of PPCPs would cause trepidation in maintaining the homeostasis of the ecological environment [27]. Thus, it is of great importance to remove PPCPs from the environment. In recent decades, the number of publications on PPCPs had been increased annually (Figure 2).

**Figure 3:**
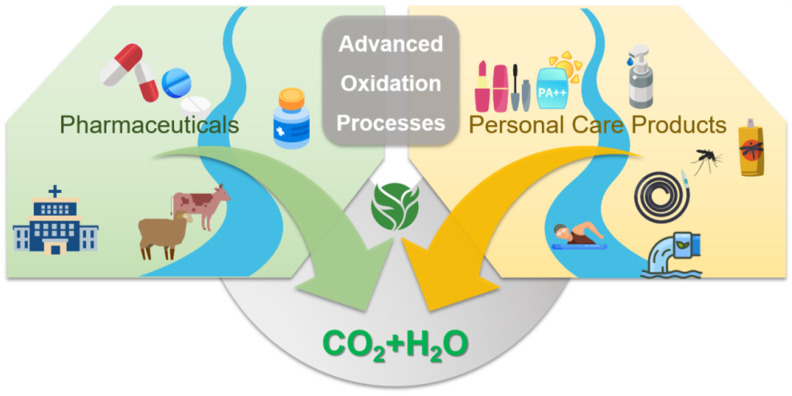
The illustration of the removal of PPCPs by free radicals in AOPs.

Wastewater treatment technologies, such as sedimentation [36], adsorption, biodegradation, sand filtration and membrane separation [37], have shown excellent removal performance in terms of conventional contaminants. Nevertheless, the removal performances of PPCPs are not satisfactory yet due to the high biotoxicity and pseudo-persistence properties of PPCPs. Most of the PPCPs were discharged to surface water after treated by municipal sewage treatment plants [38,39]. To remove PPCPs from aqueous environments, efficient approaches need to be explored. Advanced oxidation processes (AOPs), as deep treatment technologies, are acknowledged as among the most promising technologies in terms of the removal of PPCPs [40,41,42,43]. In recent decades, the number of publications focused on AOPs has grown exponentially (Figure 2). For instance, Somathilake et al. [44] used the UV-assisted ozone oxidation process for the removal of CBZ in treated domestic wastewater. The complete removal efficiency was achieved in the beginning as 0.5 min, which yielded an excellent mineralization extent of CBZ. In the process of oxidation, the degradation efficiencies of PPCPs are realized mostly by the strong oxidization of free radicals, which are in situ generated during the reaction processes under appropriate conditions such as high temperature, high pressure, microelectricity, ultrasound, light irradiation and catalysts [45,46,47]. According to different mechanisms, AOPs could be classified into photochemical oxidation [48,49], catalytic wet oxidation [50,51], ultrasonic oxidation [52,53], ozone oxidation [54,55], electrochemical oxidation [56,57], Fenton oxidation [58,59], persulfate-based oxidation [60,61], radiation oxidation [62,63], photoelectrocatalysis [64,65], etc. Due to their high reaction rates, inducible chain reactions and final products, AOPs could effectively remove PPCPs and decompose them into micromolecule compounds from complex environments (Figure 3) [66]. In AOPs, different free radicals are generated in different processes, such as hydroxyl radical (HO·), sulfate radical (SO_4_·^−^), superoxide radical (O_2_·^−^), and chlorine radical (Cl·).

Herein, the removal performance of PPCPs of different free radicals generated in various AOPs were analyzed and summarized systematically by in-depth analysis of the reaction mechanisms. Current status, future directions, perspectives and challenges of AOPs were further discussed.

## 2. Various AOPs

**Figure 4:**
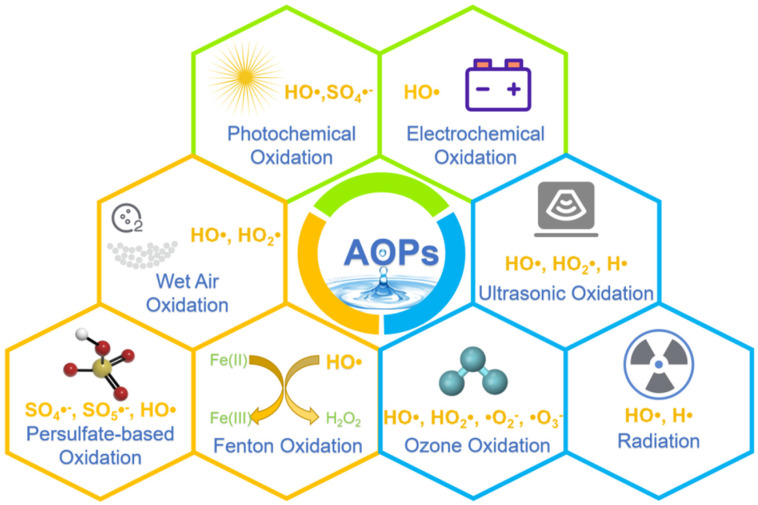
Possible free radicals generated in different AOPs.

AOPs is the general term for the photochemical oxidation process, the electrochemical oxidation process, the wet air oxidation process, the ultrasonic oxidation process, the gamma ray/electron beam radiation process, the Fenton oxidation process, the ozone oxidation process, the persulfate-based oxidation process, etc. AOPs are employed either independently or in combination with other chemical processes for the removal of PPCPs. Prior to revealing the degradation mechanism of PPCPs, it is needed to analyze and summarize the generation of possible free radicals in different AOPs. Figure 4 summarized the possible free radicals generated in different AOPs.

### 2.1. Photochemical Oxidation

Photochemical oxidation processes can be achieved by two approaches—the photocatalytic oxidation method and the photo-excited oxidation method. The former uses a semiconductor (such as TiO_2_ [67,68] and WO_3_ [69,70]) as a photocatalyst. When the semiconductor makes contact with water, strongly oxidizing free radicals (i.e., HO·) are generated on its surface (as shown in Equations (1) and (2) [71,72]), which react with PPCPs and degrade them in water. As among the most promising photocatalyst materials, nano-TiO_2_ is highly utilized to remove PPCPs from water, and nano-TiO_2_ has been studied by numerous studies [73,74,75].

TiO_2_ + hv → e_CB_^−^ + h_VB_^+^ (1)

h_VB_^+^ + OH^−^ → HO· (2)

The latter method aims at enhancing the oxidation potential of oxidants under ultraviolet (UV) irradiation. In the process, free radicals with strong oxidizing properties such as O_2_·^−^ and HO· are generated by different oxidants [76]—during which, when H_2_O_2_ and peroxysulfate are employed as oxidants separately, HO· and SO_4_·^−^ are the main free radicals, respectively (Equations (3) and (4)) [77,78]. Otherwise, when peroxymonosulfate (PMS) is employed as an oxidant (Equation (4)), both HO· and SO_4_·^−^ are the main free radicals (Equation (5)) [78].

H_2_O_2_ + hv → 2 HO· (3)

S_2_O_8_^2−^ + hv → 2 SO_4_·^−^ (4)

HSO_5_^−^ + hv → HO· + SO_4_·^−^ (5)

New insights were provided by these innovative methods for the potential application of photochemical oxidation in water pollution remediation. For instance, improving the structure and properties of catalysts would have an impact on the generation of free radicals, and further on the removal of PPCPs. Fu et al. [79] investigated a three-dimensional core-shell composite material for PPCPs degradation, which exhibited a >90% removal efficiency of CBZ. Wang et al. [80] synthesized Sr@TiO_2_/UiO-66-NH_2_ heterostructures as a photocatalyst for the degradation of ACE. In the report, strontium titanate was used as a precursor to obtain the heterostructures, resulting in the anchoring and dispersing of Sr@TiO_2_ on the surface of UiO-66-NH_2_. The process exhibited an excellent removal rate towards ACE (over 90%). This method provides a new platform for the application of MOF materials in photocatalytic oxidation processes.

With mild reaction conditions and high oxidation capacity, the photochemical oxidation process is acknowledged as an eco-friendly method. Nevertheless, it has several limitations: (1) most of the catalysts used in photochemical oxidation are nanoparticles that are difficult to recover; (2) the electron–hole pairs generated by light are easily recombined and inactivated; (3) the UV radiation has a narrow absorption range and a low utilization rate of light energy. These limitations are urgently to be improved in later research.

### 2.2. Electrochemical Oxidation

The electrochemical oxidation method is among the most acclaimed methods to remove PPCPs from aqueous environments. In the process, free radicals (i.e., HO·) are generated by the decomposition of H_2_O and simultaneously the oxidation of hydroxyl ions via direct or indirect electrochemical oxidation. The reactions were expressed in Equations (6) and (7) [78,81].

H_2_O → HO· + H^+^ + e^−^ (6)

OH^−^ → HO· + e^−^ (7)

Recently, Guo et al. [82] fabricated single-atom copper (Cu) and nitrogen (N) atom-codoped graphene (Cu@NG) and used it as electrocatalytic anode. The efficient degradation of ACE was achieved with a current of 15 mA within 90 min. Although this method was beneficial to improve the electrocatalytic performance (100% degradation efficiency), there was a lack of toxicity analysis of intermediate products. Xia et al. [83] prepared a self-made Ti/SnO_2_-Sb_2_O_3_/α,β-Co-PbO_2_ electrode to degrade norfloxacin (NOR). After electrolysis for 60 min, the removal efficiency of NOR, chemical oxygen demand (COD) and total organic carbon (TOC) were 85.29%, 43.65% and 41.89%, respectively. However, as the results revealed, in the processes of decomposing PPCPs, the toxic intermediates which perhaps harm the environment might be produced because of the low TOC removal efficiency. Therefore, later studies are expected to investigate how to achieve high efficiency of TOC.

Due to the simple assembly, easy operation and convenient control, the electrochemical oxidation device provides a facile approach to large-scale application. Nonetheless, satisfactory decomposition of PPCPs may need high energy consumption and high equipment cost, and there is a shortage of electrochemical oxidation that needs to be further solved. Furthermore, the preparation of inexpensive and efficient electrode materials for practical engineering applications is also needed.

### 2.3. Wet Air Oxidation

Wet air oxidation (WAO) uses O_2_ as an oxidant at high temperature (473–593 K) and high pressure (20–200 bar) to generate free radicals (i.e., HO·) [84]. A radical chain reaction is involved in the process of WAO [85]. In general, alkyl radical (R·) and hydroperoxide radicals (HO_2_·) are generated by the reaction of PPCPs and O_2_, which is known as a chain induced reaction. The reaction was expressed in Equation (8) [78]. HO_2_· can be converted to HO· via the chain transfer reaction, as shown in Equations (9) and (10).

RH + O_2_ → R· + HO_2_· (8)

2 HO_2_· → H_2_O_2_ + O_2_ (9)

HO_2_· + H_2_O_2_ → HO· + H_2_O + O_2_ (10)

Zhu et al. [86] employed the WAO method to treat antibiotic wastewater, with the optimum TOC removal reached being 87.3%. Likewise, Boucher et al. [87] used the WAO method as a pretreatment technology in the removal of pharmaceuticals from hospital effluents. Significant removal rates (>90%) were achieved for all pharmaceuticals under 300 °C within 60 min. This experiment indicated the potential application of WAO to remove pharmaceuticals from hospital wastewater, which could effectively prevent the release of pharmaceuticals into surface water.

The WAO method has the advantages of eco-friendliness and splendid degradation performance; however, the operation conditions are harsh, which leads to high cost in the practical application. Thus, in further studies, inexpensive WAO technologies should be explored to realize the large-scale application for the removal of PPCPs in sewage treatment plants.

### 2.4. Ultrasonic Oxidation

The ultrasonic oxidation method is a process that applies acoustic waves with frequencies ranging from 15 kHz to 1 MHz at high temperature and high pressure to remove refractory PPCPs by oxidants (i.e., HO·) [52]. During the ultrasonic oxidation of pure water, the reaction chains progressed, as expressed in Equations (11)–(14) [88].

H_2_O + ))) → HO· + H· (11)

HO· + H· → H_2_O (12)

HO· + HO· → H_2_O_2_ (13)

H· + H· → H_2_ (14)

In the above process, additional free radicals are also generated in the gas phase when the solution is saturated with O_2_. The reactions were expressed in Equations (15)–(18) [89].

O_2_ + ))) → O + O (15)

O + H_2_O → HO· + HO· (16)

O_2_ + H· → HO_2_· (17)

HO_2_· + HO_2_· → H_2_O_2_ + O_2_ (18)

In recent research, Sierra et al. [90] presented a low-frequency ultrasonic oxidation to remove cephalexin (CPX) and doxycycline (DOX) from water, and the pharmaceuticals were completely degraded at a frequency of 40 kHz. The system had a significant effect on antibiotics elimination, which could diminish the potential risk to ecosystems. Camargo-Perea et al. [91] utilized ultrasonic oxidation to degrade seven types of pharmaceuticals with different chemical structures in distilled water environments. The highest removal of DCF was obtained at 24.4 W (a complete degradation efficiency was achieved within 30 min). To extend practical applications, experiments on various aqueous mixtures need to be explored.

The ultrasonic oxidation method has typical advantages such as no addition of chemicals and selective degradation depending on the nature of various PPCPs. Meanwhile, the ultrasonic oxidation method is simple to operate and convenient to use, and can degrade toxic PPCPs into small molecules with less toxicity or no toxicity. In addition, the ultrasonic oxidation method could be utilized as an assistive technology in combination with other AOPs to remove PPCPs from water.

However, ultrasonic degradation of wastewater is still at the laboratory stage—the degradation mechanism, reaction kinetics, reactor design and amplification of the ultrasonic degradation process have not been sufficiently studied. Moreover, it takes a lot of energy to produce ultrasonic waves. The above shortages make ultrasonic degradation difficult to realize practically in environmental remediation.

### 2.5. Gamma Ray/Electron Beam Radiation

Gamma ray/electron beam radiation can activate H_2_O molecules, and cause the ionization of plenty of H_2_O molecules simultaneously in a few seconds to generate free radicals (HO·, H·) [92,93]. The reactions were expressed in Equation (19).

8 H_2_O → 4 HO· + 2 e_aq_^−^ + 2 H· + H_2_ + H_2_O_2_ + 2 H_3_O^+^ (19)

**Figure 5:**
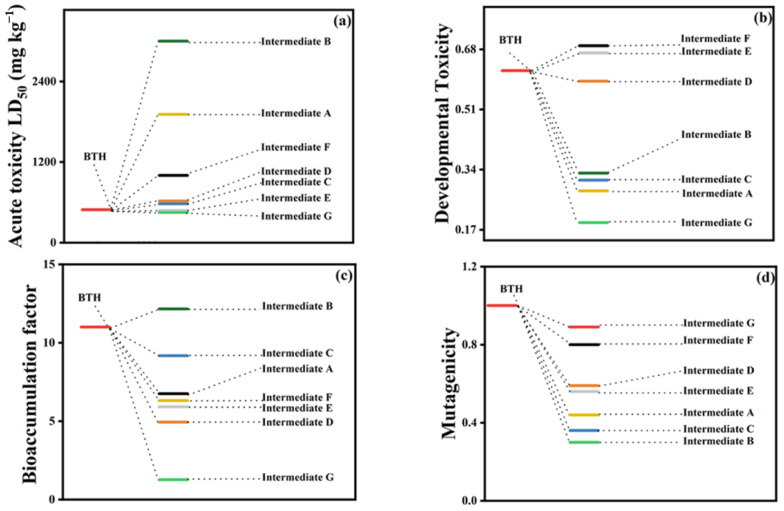
The results of the toxicity calculation of BTH and degradation intermediates in the electron beam irradiation system: (**a**) oral rat LD50; (**b**) developmental toxicity; (**c**) bioaccumulation factor; and (**d**) mutagenicity. Reproduced with permission from [92].

Chen et al. [92] used the electron beam method for the degradation of benzothiazole (BTH) in aqueous solution. Experiments showed that the method had an effective removal rate (up to 90%) towards BTH when the electron beam reaches 5 kGy. Toxicity calculations exhibited that the toxicity of most of the intermediates had been significantly reduced after radiation. As shown in Figure 5, most of the produced intermediates were non-toxic during the degradation of BTH, although there are still a few intermediates with higher toxicity than BTH. Trojanowicz et al. [93] utilized gamma ray radiation for the removal of endocrine disruptor BPA from wastewater. The degradation rate of BPA reached more than 90% in 5.5 min. These novel approaches provide new platforms for the removal of PPCPs from water.

Compared with other AOPs, the advantage of radiation processes is that it can simultaneously generate HO·, e_aq_^−^ and H· with high efficiency, which will help to achieve high degradation rates towards target contaminants. However, the defects of gamma ray radiation are also obvious, such as the requirement of a long exposure time, the regular replacement of radionuclides, and the persistent potential risk of radiation contamination.

### 2.6. Fenton Oxidation

The mixture of Fe^2+^ and H_2_O_2_ is defined as Fenton’s reagent [94]. The core process of Fenton oxidation is the reaction of Fe^2+^ and H_2_O_2_—during which, H_2_O_2_ is activated by Fe^2+^ to generate free radical HO· and HO_2_·, which are performed as the primary product and the secondary product, respectively (Equations (20)–(22)) [78,94].

Fe^2+^ + H_2_O_2_ → Fe^3+^ + HO· + HO^−^ (20)

H_2_O_2_ + HO· → H_2_O + HO_2_· (21)

2 HO· → H_2_O_2_ (22)

In recent research, the removal of CBZ and CAF from tap water by the Fenton oxidation process was studied by Sönmez et al. [95]. The results showed that the removal efficiencies of CBZ and CAF were calculated as 99.77% and 99.66%, respectively. The optimum performance was exhibited when the concentrations of H_2_O_2_ and Fe^2+^ were either 0.6 mg H_2_O_2_/L corresponding to 8 mg Fe^2+^/L or 7.5 mg H_2_O_2_/L corresponding to 6 mg Fe^2+^/L. The reaction conditions of Fenton oxidation are mild without high temperature or high pressure, and the device is also easy to operate independently or combined with other treatment technologies. However, there are several disadvantages of Fenton oxidation, such as the limit of the acidic condition and the production of a large amount of iron-containing sludge which is difficult to remove [96]. To overcome these disadvantages, Fe-based porous catalysts are used to replace the role of the dissolved ion Fe^2+^, to get access to recycle, based on which the process are called as Fenton-like oxidation process, including photo-Fenton oxidation and electro-Fenton oxidation [97]. In the above Fenton-like oxidation processes, the removal of PPCPs in aqueous solution can be achieved within an expanded applicable pH range, and the degradation performance is also superior to that of the classical Fenton oxidation process.

### 2.7. The Ozone Oxidation Process

The ozone oxidation process is a widely used AOP in the removal of PPCPs. Ozone has high oxidizing ability with a redox potential of 2.07 eV [98]. In the process of ozone oxidation, HO·, O_2_·^−^, O_3_·^−^ and HO_2_· are generated by chain reactions, as shown in Equations (23)–(27) [99], which can decompose recalcitrant PPCPs efficiently.

O_3_ + OH^−^ → HO_2_^−^ + O_2_ (23)

3 HO_2_^−^ + 2 O_3_ → 3 O_2_·^−^ + 3 HO_2_· (24)

HO_2_· → O_2_·^−^ + H^+^ (25)

O_2_·^−^ + O_3_ → O_3_·^−^ + O_2_ (26)

O_3_·^−^ + H_2_O → HO· + O_2_ + OH^−^ (27)

Yang et al. [100] employed a catalytic ozone oxidation/membrane filtration process to degrade SMX in aqueous environments. The catalyst was prepared by the method of impregnation combined with in situ precipitation. The degradation efficiency was up to 81.3% after the treatment of the oxidation–filtration combined process, which provides new ideas for the combination of AOPs and other technologies. Paucar et al. [101] investigated the degradation performance of the ozone oxidation process towards PPCPs in the secondary effluent of a sewage treatment plant. The results exhibited that the ozone oxidation process is absolutely capable of decomposing a wide range of PPCPs in 10–15 min. These attempts provide novel methods for future studies [102,103,104].

The ozone oxidation process is a promising technology to decompose PPCPs from water environments, which has no secondary pollution. However, to date, there is no consensus on the mechanisms of the ozone oxidation process, and this needs further investigation. Meanwhile, the low utilization efficiency of ozone is also a significant issue for further water treatment applications.

### 2.8. Persulfate-Based Oxidation

**Figure 6:**
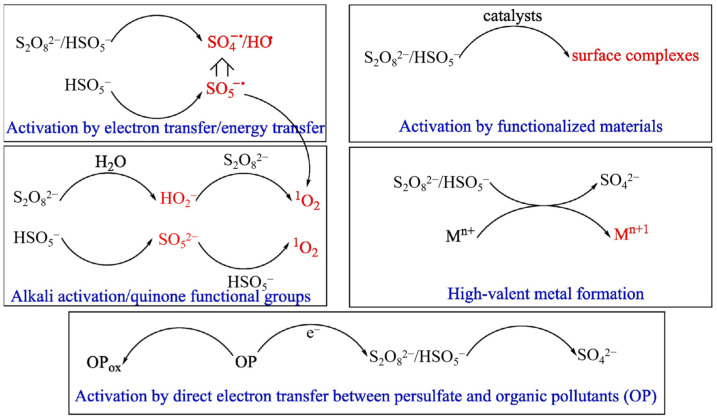
Possible activation mechanisms of persulfate. Reproduced with permission from [78].

Persulfate, including PDS (peroxydisulfate, S_2_O_8_^2−^) and PMS (HSO_5_^−^), can be activated to produce free radical SO_4_·^−^, which has the characteristic of strong oxidation. Commonly, the persulfate can be activated by methods including thermal activation [105,106], mechanochemical activation [107,108], carbonaceous materials activation [109,110], alkali activation [111,112], electrochemical activation [113,114], UV activation [115,116], and transition metal activation [117,118]. The possible activation mechanisms were shown in Figure 6. Generally, in addition to SO_4_·^−^, free radicals including SO_5_·^−^ and HO· can also be generated during various processes of persulfate activation [119,120].

In a recent study [121], the heat-activated PMS process was introduced to remove ACE from water environments, during which sodium tetraborate was used as a catalyst. The results indicated that the degradation efficiencies of ACE were significantly high (nearly 100%) in multiple mediums including ultrapure water, lake water and groundwater within 15 minutes of reaction time. Weng et al. [122] utilized Fe^0^ to activate the persulfate oxidation system via hydrodynamic cavitation. The removal rate of tetracycline (TC) was up to 97.80%. These studies have shed light on the potential implementation of the persulfate oxidation process on the removal of PPCPs.

Despite the fact that the persulfate-based oxidation process has been widely reported, it is still at the experimental stage. The industrial application needs to be studied thoroughly. Furthermore, later studies on persulfate-based oxidation processes should focus on evaluating their comprehensive performance, i.e., activation rates of persulfate, energy cost, toxicity and yield of by-products.

**Figure 7:**
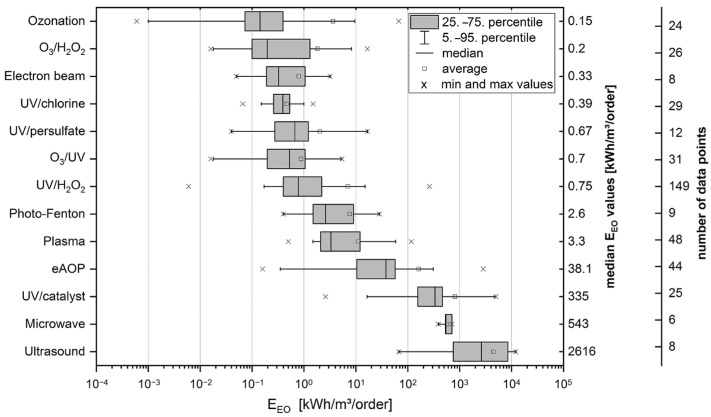
Overview of published EEO values of different AOPs sorted according to median values. For O_3_- and UV-based AOP data, only substances resistant to direct ozonation/photolysis are shown. Median values and number of data points are reported on the second and third y-axis, respectively. Reproduced with permission from [123].

To evaluate the electrical energy per order (E_EO_) values of various AOPs, significant differences among AOP efficiency were observed by Miklos et al. [123]. As shown in Figure 7, based on reported E_EO_ values, AOPs were classified into (1) AOPs with median E_EO_ values of <1 kWh/m^3^, (2) processes with median E_EO_ values in the range of 1–100 kWh/m^3^ and (3) median E_EO_ values of >100 kWh/m^3^ (i.e., UV-based photocatalysis, ultrasound, and microwave-based AOPs), which are considered as not (yet) energy efficient AOPs. The research provides an excellent figure of merit to directly compare and evaluate AOPs based on energy efficiency.

Overall, to efficiently remove PPCPs from aqueous environments, there has been a lot of effort to develop different types of AOPs or their combined technologies. Table 2 summarized the dominant free radicals, experimental conditions, and removal efficiencies of different processes on the removal of several PPCPs with the highest detection frequencies. However, among all the developed AOPs, there is still no process which can simultaneously achieve the goals of high efficiency, low cost and simple operation on the removal of PPCPs. Thus, further studies should focus on combining different types of free radicals and explore more advanced processes to make AOPs more considerable for industrial application.

## 3. Different Free Radicals in the Removal of PPCPs

### 3.1. Hydroxyl Radical (HO·)

**Figure 8:**
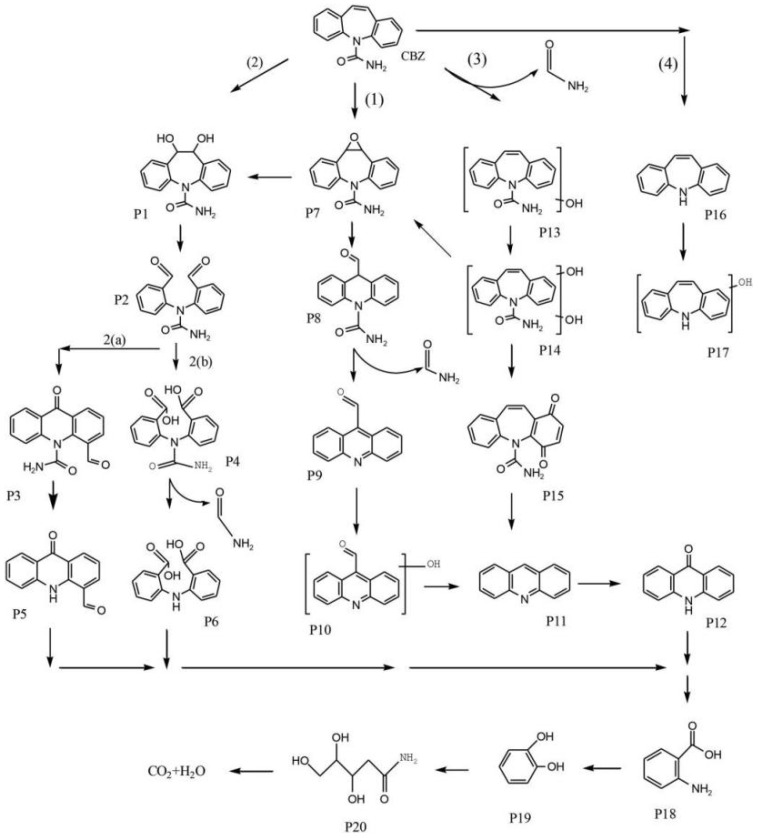
Degradation pathway of CBZ in the EF process with FeS_2_/CF as a cathode. Reproduced with permission from [145].

Free radical HO· has a redox potential of 2.80 V and extremely strong oxidizing potential [142]. In AOPs, the HO· is mainly generated via hydrogen abstraction and hydroxylation [143]. The HO· is a strong oxidant which can react with the unsaturated carbon-carbon bond, the carbon-nitrogen bond, the carbon-sulfur bond and other chemical bonds to decompose PPCPs, with non-selective chemical oxidation [144]. The Fenton oxidation process is a typical AOP, which generates HO· by chain reactions between H_2_O_2_ and Fe^2+^ under acidic conditions. Nevertheless, the Fenton oxidation process has the disadvantages of using excessive amounts of Fe^2+^ and H_2_O_2_, which resulted in low utilization of H_2_O_2_. Compared to the conventional Fenton oxidation process, the required H_2_O_2_ could be in situ generated by the electro-Fenton process, which saves cost and improves decomposition efficiency. Currently, Cui et al. [145] designed an electro-Fenton (EF) oxidation device to degrade carbamazepine (CBZ) in water. In the report, FeS_2_/carbon felt was used as the cathode and Ti/IrO_2_-RuO_2_ was used as the anode of the EF device. The reaction between H_2_O_2_ and Fe^2+^ was accelerated by the cathode, which helped to produce more HO· to remove CBZ. The possible degradation pathways of CBZ were shown in Figure 8. Under the attack of HO·, CBZ molecules were finally mineralized and decomposed into harmless and small molecules with a degradation rate of 99.99%.

**Figure 9:**
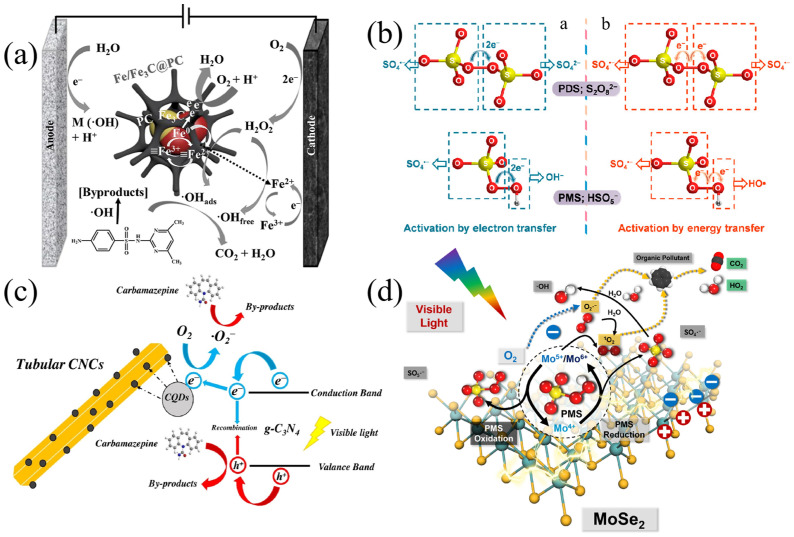
Illustrations of mechanisms of different AOPs. (**a**) Proposed scheme of the mechanism of the hetero-EF process catalyzed by Fe/Fe_3_C@PC. Reproduced with permission from [146]. (**b**) Activation of PDS and PMS through **a** electron transfer and **b** energy transfer reactions. Reproduced with permission from [149]. (**c**) Schematic illustration of the photocatalytic mechanism using carbon quantum dots modified tubular graphitic carbon nitride as material. Reproduced with permission from [150]. (**d**) Possible mechanisms of the visible light-driven MoS_2_/PMS system. Reproduced with permission from [151].

Du et al. [146] investigated Fe/Fe_3_C@PC hybrid materials with core-shell structures as catalysts for the degradation of sulfadimethoxine (SMT) in the non-homogeneous Fenton process. The MIL-101(Fe) precursor was prepared by the solvothermal method. Then, the activated precursor was put in a tube furnace under a flowing argon atmosphere and heated to 800 °C for 6 h at a heating rate of 5 °C min^−1^ to produce Fe/Fe_3_C@PC. The removal of SMT was up to 96% at pH = 4 with a microcurrent of 25 mA. Furthermore, the degradation rate decreased with the increase in pH, due to decomposition into H_2_O and O_2_ when pH ≥ 4.5, which hindered the generation of HO· [147]. The oxidation potential of HO· decreased with the increase in pH (E^0^ = +2.8 V at pH = 0 and E^0^ = +1.98 V at pH = 14) [148]. As shown in Figure 9a, the dominant free radical was HO·. Density functional theory (DFT) calculations indicated the presence of internal microelectrolysis (IME) in Fe/Fe_3_C@PC hybrid materials has greatly promoted the activation of H_2_O_2_ to generate HO·. During the electrolysis process, SMT molecules were attacked by HO· and ultimately decomposed into innocuous CO_2_ and H_2_O.

In summary, HO·-based AOPs can effectively remove PPCPs from water environments. Nevertheless, there are still some difficulties in HO· radicals-based AOPs. On the one hand, HO·-based AOPs have several limitations. For instance, to the best of our knowledge, HO·-based AOPs have the disadvantages of a large amount of reagents, no selectivity towards target substances, and narrow pH conditions (pH 3~4). Therefore, further optimization and exploration of degradation conditions are expected. On the other hand, the identification of HO· is significantly imprecise. Recently, Chen et al. [152] found that in the UV-based AOPs, the widely used scavenger alcohol will accidentally generate H_2_O_2_ in the process of eliminating HO·. These generated H_2_O_2_ will be photodissociated into HO·, thus affecting the accuracy of HO· quantification. Therefore, researchers should select suitable scavenger to eliminate HO· like *N*-butyl alcohol, and more accurate identification technologies should be proposed and promoted. Lastly, the HO·-based AOPs are still at the experimental research stage, when it comes to industrial application, existing problems such as high operation cost need to be solved. In the later research, how to realize practical application and improve the selectivity oxidation of target PPCPs are the current challenges of Fenton-like technologies. Meanwhile, efficient and stable catalysts should be developed to increase utilization efficiency and reduce energy cost.

### 3.2. Sulfate Radical (SO_4_·^−^)

Studies on sulfate radical (SO_4_·^−^) for pollutants removal began in 1996 [153]. Compared to HO·, SO_4_·^−^ has a higher average redox potential (E^0^ = 2.5–3.1 V), a wider pH range (2–10) and a longer half-life (30–40 μs) [154]. The decomposition of SO_4_·^−^ towards PPCPs was mainly achieved via electron transfer [155,156]. By activation through electron transfer, PDS or PMS molecules are converted to SO_4_·^−^ [149,157]. Generally, persulfates are the main precursors of SO_4_·^−^ (Figure 9b) [47,149]. Compared with the process without PDS, the decomposition efficiency of organic pollutants in the process with PDS was greatly accelerated [47,158]. In this system, the procedure of persulfate activization was a crucial process in AOPs. Theoretically, most of the organic matters in water environments could be removed by the oxidation of SO_4_·^−^ [159]. Zhang et al. [160] used carbon nanofiber-loaded Co/Ag bimetallic nanoparticles (Co@CNFS-Ag) as catalysts for the heterogeneous activation of PMS and the efficient oxidation of amoxicillin (AMX). The excellent removal performance of AMX was realized by adjusting the dosage of catalyst, the reaction temperature and the pH condition. The results indicated that the optimal pH of this system was 7, which was environmentally friendly.

Affected by impurities and other interference, the degradation of some PPCPs in the actual water environments by SO_4_·^−^ may produce intermediates, byproducts and residues that are even more toxic and difficult to degrade. Thus, the utilization of SO_4_·^−^-based AOPs needs to be further improved to eliminate their negative impacts. In addition, in the quenching identification process of SO_4_·^−^, the high concentration of added scavengers would cause numerous confounding effects on the persulfate-based process, thus affecting the generation of SO_4_·^−^. Therefore, adding scavengers may seriously mislead the interpretation of the mechanism of SO_4_·^−^-based AOPs [161]. Thus, the mechanism of adding scavengers to explain SO_4_·^−^-based AOPs should be cautious, and some controversial conclusions obtained by adding scavengers may need to be re-examined.

### 3.3. Superoxide Radical (O_2_·^−^)

Recently, superoxide radical (O_2_·^−^) has attracted increasing concern in environmental remediation because of its potential to destroy highly toxic organic chemicals which are carcinogenic in most cases [162]. The redox potential of O_2_·^−^ is 2.4 V. O_2_·^−^ can induce the degradation of PPCPs through an initial hydrogen abstraction step, which results in the formation of carbon-based radicals [163,164]. Then, carbon-based radicals combine with O_2_ to form peroxide intermediates. Afterwards, the formation of degradation products was realized [165]. O_2_·^−^ is an important species involved in natural aquatic systems exposed to sunlight [166].

**Figure 10:**
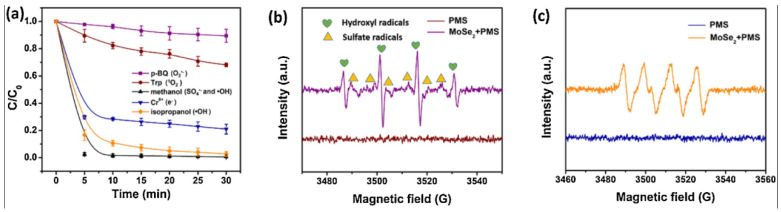
(**a**) Scavenger test for the degradation of CBZ; (**b**) EPR spectra for the detection of HO· and SO_4_·^−^ in the presence of 5,5-dimethyl-1-pyrrolidineN-oxide (DMPO) at room temperature; (**c**) EPR spectra for the detection of O_2_·^−^ in the presence of DMPO and methanol, (CH_3_OH) at room temperature. Reproduced with permission from [151].

Photocatalysis, an AOP, has been shown to be an effective method for the generation of O_2_·^−^. Zhao et al. [150] prepared novel carbon quantum dots (CQDs)-modified tubular graphitic carbon nitride (g-C_3_N_4_) by an adsorption–polymerization method, which showed up to a 100% removal rate towards CBZ under visible light irradiation. It was further confirmed by electron spin resonance (ESR) analysis that the main active species for CBZ degradation were O_2_·^−^ and photogenerated holes (h^+^). The detailed mechanism was shown in Figure 9c. Under the attack of O_2_·^−^ and h^+^, CBZ molecules were mineralized to harmless CO_2_ and H_2_O. Dong et al. [151] established a visible light-driven PMS activation process dominated by O_2_·^−^. In the study, the generation of radicals was confirmed via combination with the scavenger test and electron paramagnetic resonance (EPR) detection. During the scavenger test, HO· and SO_4_·^−^ were captured by methanol, HO· was captured by isopropanol, O_2_·^−^ was captured by p-BQ, and the results indicated that HO·, SO_4_·^−^ and O_2_·^−^ were all generated in this system, among which O_2_·^−^ played a dominant role (Figure 10a). To further verify the generation of free radicals, EPR was employed to detect these free radicals, coupled with 5,5-dimethyl-1-pyrroline (DMPO) as a spin-trapping reagent to capture both SO_4_·^−^ and HO·. The intensity of characteristic peaks for DMPO·-SO_4_^−^ and DMPO·-HO was observed (Figure 10b), verifying the existence of SO_4_·^−^ and HO·. As shown in Figure 10c, after the addition of methanol and DMPO, the characteristic peaks of DMPO·-O_2_^−^ were observed, confirming the generation of O_2_·^−^. The possible mechanism was exhibited in Figure 9d. Once irradiated with visible light, the charge carriers (i.e., electrons and holes) were generated on the surface of MoSe_2_ (Equation (28)). Additionally, photo-generated electrons react with O_2_ to produce O_2_·^−^ (Equation (29)). Due to the generation of photoelectrons, IBP, benzophenone-3 (BZP) and CBZ were decomposed in aqueous environments (Equation (30)). These studies on the generation and identification of O_2_·^−^ could provide mechanisms and theoretical bases for understanding the comprehensive processes of photocatalysis.

MoSe_2_ + hv→e^−^ + h^+^ (28)

O_2_ + e^−^ → O_2_·^−^ (29)

O_2_·^−^ + target PPCP → intermediates + CO_2_ + H_2_O (30)

In photocatalysis reactions, O_2_·^−^ is an important reactive oxygen species. The study of the generation and presence of O_2_·^−^ could help to promote the understanding of the photocatalysis mechanism. Moreover, it could provide a guideline and theoretical basis for improving photocatalysis efficiency.

### 3.4. Reactive Chlorine Species (RCS)

The reactive chlorine species (RCS) of the UV/chlorine process is an emerging AOP used for the removal of PPCPs [167]. RCS (including Cl·, Cl_2_·^−^ and ClO·^−^) were found to exhibit excellent removal rates towards many types of PPCPs including chlorine-resistant and UV-resistant PPCPs, i.e., CBZ and CAF [168]. Compared with HO·, Cl· has a high redox potential (2.5 V) as well as high selectivity [169]. Cl· can degrade PPCPs by the reactions of hydrogen abstraction, one-electron oxidation and chlorine addition [170]. As known, the UV/chlorine process is a more effective technology to remove PPCPs (i.e., CBZ, sulfamethoxazole and IBP) than the UV/H_2_O_2_ process for the reason that more effective free radicals are generated in the former process. In addition, the residual chlorine could be used for water disinfection in the former process. Thus, the UV/chlorine process could be considered as a possible alternative to the UV/H_2_O_2_ process for water treatment plants. Xiang et al. [171] investigated the degradation kinetics and pathways of IBP in the UV/chlorine process. In the same reaction condition, the primary rate constant of in UV/chlorine process was 3.3-fold higher than that of the UV/H_2_O_2_ process. Guo et al. [172] used a UV/chlorine process to treat various types of different PPCPs. Experimental results showed that HO·, Cl·, Cl_2_·^−^ and ClO·^−^ were generated in the process. The concentration of HO· decreased significantly with the increase in pH, while the concentration of ClO·^−^ decreased gradually, and the concentration of ClO·^−^ remained essentially constant. The concentration of ClO·^−^ was 3–4-fold higher than that of HO·, Cl· or Cl_2_·^−^, so ClO·^−^ played a key role in the effective removal of PPCPs. In summary, the UV/chlorine process provides a new idea for the removal of PPCPs from waters.

It is worth noting that although the UV/chlorine process is more effective than the UV/H_2_O_2_ process, the toxicity of chlorinated products needs to be further evaluated. Meanwhile, when it comes to practical application, the high requirement of equipment and relatively high cost are also crucial issues which need to be further evaluated.

In addition to the above-mentioned free radicals, secondary radicals such as Br·, Br_2_·^−^, ClBr·^−^, and CO_3_·^−^ generated during the UV/chlorine process also contribute to promote the removal rates of PPCPs in waste water [173,174,175,176,177,178].

In future studies, the AOPs with different types of free radicals could be combined to achieve efficient removal towards PPCPs under facilitated and environmentally friendly conditions, to make it acceptable for large-scale industrial applications finally. Recently, Wang et al. [179] established a novel AOP of bisulfite (BS)/chlorine dioxide (ClO_2_) concomitant system, and the removal efficiency of atrazine (ATZ) in water was more than 85% within 3 min. In the above process, the BS was activated by ClO_2_. The scavenger experiments and ESR detection results indicated that the dominant radicals were ClO·^−^ and SO_4_·^−^. Cheng et al. [180] used a combination process of solar irradiation and free available chlorine (FAC) to remove PPCPs from drinking water. It was found that that the in situ generation of HO·, RCS and ozone by FAC under solar irradiation contributed greatly to the degradation of PPCPs. PPCPs containing electron-donating groups were degraded more rapidly and preferentially by RCS and/or HO·. Paracetamol, IBP and ATZ that contain electron-withdrawing groups were degraded mainly by HO·. The combination of solar irradiation and FAC was well-established and inexpensive, which provided a novel idea for the combination of AOPs. In the removal of PPCPs, based on the structure and physicochemical properties of the target PPCPs, studies are expected to develop the combined activation technologies to achieve selective degradation or mineralization of PPCPs.

## 4. Perspective

This review briefly summarized multiple free radicals generated by AOPs for the removal of PPCPs and look towards the recent progress of various AOPs. In the process of contaminants remediation, AOPs have the advantages of strong oxidation potential, a rapid reaction rate and complete degradation compared to conventional oxidation processes, especially for low-concentration and recalcitrant pollutants. Thus, AOPs are acknowledged as ideal and prospecting technologies in the removal of PPCPs from practical water environments. However, AOPs have disadvantages such as high operation cost and harsh experimental conditions, which make it difficult to apply AOPs at a large scale for industrial application. To achieve efficient degradation of PPCPs, the following suggestions were made for further studies.

(1) To precisely identify the concentration of free radicals, more accurate approaches should be employed. The conventional radical identification methods are challenged because adding scavengers would cause negative effects on AOPs and influence the generation of free radicals. Other methods such as probe-based kinetic models, EPR and laser flash photolysis should be developed and employed to assist in identification of the free radicals in AOPs.
(2) To achieve the practical application of AOPs, convenient and inexpensive approaches should be studied. At present, there are still some issues with AOPs, which need to be further studied. For example, the experimental operations are still complicated and have a high cost. Thus, while researchers focus on the efficiency of water treatment, convenient and inexpensive approaches should be studied to realize large-scale application.
(3) To further study the reaction process and reveal the impacts of AOPs, the mechanism of AOPs in multiple mixture systems should be investigated in depth and the interference of impurity ions on the degradation reaction should be minimized.
(4) To remove PPCPs from complex aqueous mixtures, a pilot-scale plant on the treatment of practical wastewater should be implemented instead of a laboratory scale experiment. Currently, most studies on the removal of PPCPs are focused on water environments containing limited given substances, which is unrealistic. Thus, practical wastewater (i.e., pharmaceutical discharges) should be used in later studies.
(5) To selectively remove PPCPs from aqueous environments, AOPs should be further developed to adjust them to multiple water environments. In the later studies, the removal of PPCPs is expected to be achieved without affecting the existence of trace nutritious natural organic matters (NOMs) in water environments [181].

In addition, eco-friendly AOPs should be studied by adjusting experimental conditions, such as ultrasonic power, radiation dose, current density, temperature, pH, reaction time and chemical dosage. Future studies are advised to focus on the combination of AOPs and other available technologies to selectively and efficiently remove PPCPs from water environments in a green and environmental way.

## Figures and Tables

**Table 1:** The detection of partial PPCPs in various aquatic environments from different countries.

Location	Aquatic Environment	Chemical	Category	Concentration	Ref.
Poland	Groundwater	N,Ndiethyl-meta-toluamide(DEET)	Mosquito and insect repellants	17.28 μg/L	[28]
		17β-oestradiol	Hormones	48 ng/L	
	Surface water	CAF	Stimulants	29.9955 μg/L	
		Bisphenol A (BPA)	Hormones	3.113 μg/L	
	Drinking water	Azithromycin (AZM)	Antibiotics	193 ng/L	
		Paracetamol	Non-steroidal anti-inflammatory drugs	173 ng/L	
		IBP	Non-steroidal anti-inflammatory drugs	224 ng/L	
		CAF	Stimulants	159 ng/L	
Brazil	Surface water	Avobenzone (ABZ)	Sunscreen agents	340 ng/L	[29]
		Glibenclamide (GBC)	Hypoglycemic drugs	50–120 ng/L	
	Drinking water	Nimesulide (NI)	Non-steroidal anti-inflammatory drugs	181 ng/L	
		Methylparaben	Preservatives	234 ng/L	
		ABZ	Sunscreen agents	290 ng/L	
China	Surface water	SMX	Antibiotics	<LOQ–2.92 ng/L	[30]
		4-n-nonylphenol	Hormones	9.90–457.40 ng/L	
		Salicylic acid (SA)	Analgesics	2.92–34.12 ng/L	
	Drinking water	Sulfamethoxypyridazine	Antibacterial	107.14 ng/L	[31]
		Lincomycin	Antibiotics	1.00–29.32 ng/L	
		SMX	Antibiotics	<LOQ–2.18 ng/L	
Vietnam	Surface water	CBZ	Antiepileptics	<LOQ–57.4 ng/L	[32]
		LCM	Antibiotics	<LOQ–378 ng/L	
		SMZ	Antibiotics	3.65–2778 ng/L	
India	Groundwater	Ketoprofen (KPF)	Non-steroidal anti-inflammatory drugs	<LOQ–23.4 ng/L	[33]
		IBP	Non-steroidal anti-inflammatory drugs	<LOQ–49.4 ng/L	
		CAF	Stimulant	15.2–262 ng/L	
Pakistan	Groundwater	Tigecycline	Antibiotics	21.3 ng/L	[34]
		Ciprofloxacin (CIP)	Antibiotics	18.2 ng/L	
United States	Drinking water	CBZ	Antiepileptic	51 ng/L	[35]
		DCF	Non-steroidal anti-inflammatory drugs	1.2 ng/L	
		SMX	Antibiotics	110 ng/L	
		Naproxen	Non-steroidal anti-inflammatory drugs	32 ng/L	

**Table 2:** Degradation compilation of frequently detected PPCPs via various AOPs.

PPCP	AOPs	Dominant Radicals	Concentration of PPCPs	Reaction Time (min)	pH	Removal Rate (%)	Ref.
CBZ	Photochemical oxidation	HO·, O_2_·^−^	8.75 mg/L	45	6	99.77	[124]
	Persulfate-based + photochemical oxidation	SO_4_·^−^, HO·, O_2_·^−^	5 ppm	30	7	97.1	[125]
	UV/chlorine oxidation	HO·, Cl·	40 μM	60	7	83.9	[126]
	Electrochemical oxidation	HO·, SO_4_·^−^	1 μM	5	2	100	[127]
	Photo Fenton oxidation	HO·	285 ng/L	30	6–7	94	[128]
	Photo-assisted ozone oxidation	HO·	5 mg/L	2	7 ± 0.2	>99.99	[44]
	Sono-photocatalytic oxidation	O_2_·^−^	10 ppm	240	7	93.37	[129]
	Electron beam radiation	HO·, H·	75 mg/L	/	6.3	99.9	[130]
DCF	Photochemical oxidation	HO·	20 mg/L	270	5	76	[131]
	Sonoelectrochemical oxidation	HO·	50 μg/L	5	4	96.8	[132]
	Fenton-like oxidation	HO·	20 mg/L	60	5	86.62	[133]
	Ozone oxidation	HO·	29.6 mg/L	60	7	73.3	[134]
	Persulfate-based oxidation	SO_4_·^−^	20 mg/L	15	5	90	[135]
SMX	Photochemical oxidation	HO·, O_2_·^−^	10 mg/L	60	7.5	84	[136]
	Electrochemical oxidation	HO·	74.45 mg/L	51.49	4.78	100	[137]
	Ultrasonic oxidation	HO·	10 μM	60	7	97	[138]
	Photo-Fenton oxidation	HO·	25 mg/L	15	3	98.06	[139]
	Persulfate-based oxidation	SO_4_·^−^	0.04 mM	120	3.4	100	[140]
	Gamma ray radiation	HO·	39.48 μM	/	6.7	88.6	[141]

## Data Availability

Not applicable.
